# Combining Single-Cell and Transcriptomic Data Revealed the Prognostic Significance of Glycolysis in Pancreatic Cancer

**DOI:** 10.3389/fgene.2022.903783

**Published:** 2022-07-05

**Authors:** Liang Chen, Yunhua Lin, Wei Wei, Yue Wang, Fangyue Li, Wang Du, Zhonghua Yang, Yiming Hu, Xiaomei Ying, Qikai Tang, Jiaheng Xie, Hongzhu Yu

**Affiliations:** ^1^ Department of General Surgery, Fuyang Hospital Affiliated to Anhui Medical University, Fuyang, China; ^2^ The First Clinical Medical College, Guangxi Medical University, Nanning, China; ^3^ Department of Pathology, School of Basic Medical Sciences, Anhui Medical University, Fuyang, China; ^4^ College of Pharmacy, Jiangsu Ocean University, Lianyungang, China; ^5^ Department of General Surgery, Suzhou Hospital of Anhui Province, Suzhou, China; ^6^ Department of Neurosurgery, The First Affiliated Hospital of Nanjing Medical University, Jiangsu Province Hospital, Nanjing, China; ^7^ Department of Burn and Plastic Surgery, The First Affiliated Hospital of Nanjing Medical University, Jiangsu Province Hospital, Nanjing, China

**Keywords:** pancreatic cancer, glycolysis, single-cell, immune infiltration, prognosis

## Abstract

**Background:** Pancreatic cancer (PC), the most common fatal solid malignancy, has a very dismal prognosis. Clinical computerized tomography (CT) and pathological TNM staging are no longer sufficient for determining a patient’s prognosis. Although numerous studies have suggested that glycolysis is important in the onset and progression of cancer, there are few publications on its impact on PC.

**Methods:** To begin, the single-sample gene set enrichment analysis (ssGSEA) approach was used to quantify the glycolysis pathway enrichment fraction in PC patients and establish its prognostic significance. The genes most related to the glycolytic pathway were then identified using weighted gene co-expression network analysis (WGCNA). The glycolysis-associated prognostic signature in PC patients was then constructed using univariate Cox regression and lasso regression methods, which were validated in numerous external validation cohorts. Furthermore, we investigated the activation of the glycolysis pathway in PC cell subtypes at the single-cell level, performed a quasi-time series analysis on the activated cell subtypes and then detected gene changes in the signature during cell development. Finally, we constructed a decision tree and a nomogram that could divide the patients into different risk subtypes, according to the signature score and their different clinical characteristics and assessed the prognosis of PC patients.

**Results:** Glycolysis plays a risky role in PC patients. Our glycolysis-related signature could effectively discriminate the high-risk and low-risk patients in both the trained cohort and the independent externally validated cohort. The survival analysis and multivariate Cox analysis indicated this gene signature to be an independent prognostic factor in PC. The prognostic ROC curve analysis suggested a high accuracy of this gene signature in predicting the patient prognosis in PC. The single-cell analysis suggested that the glycolytic pathway may be more activated in epithelial cells and that the genes in the signature were also mainly expressed in epithelial cells. The decision tree analysis could effectively identify patients in different risk subgroups, and the nomograms clearly show the prognostic assessment of PC patients.

**Conclusion:** Our study developed a glycolysis-related signature, which contributes to the risk subtype assessment of patients with PC and to the individualized management of patients in the clinical setting.

## Introduction

Pancreatic cancer (PC) is one of the most aggressive malignant solid tumors, and it remains the fourth leading cause of cancer-related deaths worldwide, with an overall survival rate of less than 5% (Duan et al., 2018; Ye et al., 2020). Worldwide, hundreds of thousands of new patients are diagnosed with PC each year, and nearly 200,000 people die from the disease (Abel and Simeone, 2013; McGuire, 2016). In PC, CA19-9 (a carcinoembryonic antigen) is approved by the FDA for prognostic monitoring in patients with known PC; however, it is considered having low sensitivity and specificity for PC detection (Eissa et al., 2019). Moreover, the clinical prognosis of patients cannot be accurately evaluated by the TNM staging system and imaging CT and MRI (Allenson et al., 2017). Therefore, it is particularly important to find novel prognostic markers.

Glycolysis operates under aerobic and anaerobic conditions to produce pyruvate. Tumors have long been known to be involved in aerobic glycolysis (Quinn et al., 2020). Recent studies have found that glycolysis plays an important role in the development of cancer and is mainly associated with cell proliferation, angiogenesis, and migration (Cascone et al., 2018; Zhong et al., 2020), especially in hepatocellular carcinoma (HCC), triple-negative breast cancer (TNBC), colorectal cancer (CRC), and lung cancer (Guo et al., 2021; Shen et al., 2021; Wang et al., 2021; Xie et al., 2021). It was found that the pancreatic tumors may rely heavily on glycolysis (Qin et al., 2019; Yang et al., 2019), but the prognostic impact of glycolysis-related genes on PC patients and the activation of this pathway in PC cell subtypes have not been fully studied.

In our study, we first investigated the prognostic impact of glycolysis-related genes in PC. Then, we identified the most relevant genes for glycolysis by the WGCNA method and constructed a glycolysis-related prognostic signature to assess the patient prognosis. Also, this signature was validated in multiple external cohorts. Subsequently, we further investigated the glycolytic pathway and the genes in the signature at the single-cell sequencing level in PC cancer. Finally, we constructed a decision tree analysis and nomogram to identify the risk subgroups of PC patients and further facilitate personalized management of patients.

## Material and Methods

### Transcriptome Data Download and Processing Process

UCSC Xena (http://xena.ucsc.edu/) is a comprehensive website that collects and organizes sequencing data and clinical data from multiple oncology databases. In this study, a cohort (GDC TCGA pancreatic cancer [PAAD]) was downloaded from this database, including the normalized transcriptome data (HTSeq-FPKM) and the corresponding clinical data. As the M-stage of many PC patients in TCGA database could not be accurately determined, the M-stage was not included in subsequent analyses such as Cox regression analysis. The International Cancer Genome Consortium (ICGC) database collects tumor data on different cancer types or different subtypes, including gene expression data and related clinical data, etc., and is commonly used to make a comparison of the conclusions obtained from the TCGA cohort. In this study, two cohorts of pancreatic cancer (pancreatic cancer-AU [PACA-AU] and pancreatic cancer-CA [PACA-CA]) were downloaded from the ICGC database, including gene expression data and clinical data. We found that the clinical information of the PACA-AU cohort included survival time, survival status, gender, and age, and the clinical information of the PACA-CA cohort included the survival time, survival status, gender, age, and tumor stage. Then, 80 and 213 samples containing both expression and clinical data, respectively, were obtained by matching. The data are shown in Supplementary Table S1. The expression data were log2-transformed and used for subsequent analysis.

### Single-Cell Sequencing Data Download and Processing Flow

The Gene Expression Omnibus (GEO) database contains microarray data, high-throughput gene expression data, and single-cell sequencing data submitted by research institutions worldwide. In this research, a single-cell sequencing dataset of PC containing 16 samples, GSE154778, was downloaded from the GEO database. First, genes expressed in fewer than three cells were removed. The cells containing only 300 or fewer genes were then removed. Subsequently, 2,000 anchors were set for analysis using the Seurat package’s “FindIntegrationAnhors” function, and the samples were integrated using the “IntegrateData” function. Finally, the principal component analysis method was used to reduce the dimension by setting the number of principal components as 20. The results of dimensionality reduction and clustering are presented in the form of a uniform manifold approximation and projection (UMAP) graph. The “SingleR” package is mainly used to annotate the cell types such as humans and mice. In this study, the cell types were annotated synthetically by using the SingleR package and Cell Markers website.

### Single Sample Gene Set Enrichment Analysis

“ssGSEA”is implemented by extending the gene set enrichment analysis (GSEA) to allow the definition of an enrichment score that represents the degree of enrichment of each sample in a given dataset in the gene set. In this study, the glycolytic gene sets were downloaded from the GSEA website, and the ssGSEA method was used to calculate the glycolytic enrichment score for each PC sample.

### Weighted Gene Correlation Network Analysis

WGCNA is a systems biology method used to characterize the gene association patterns between the different samples and can be used to identify the highly synergistic sets of genes to identify the candidate biomarker genes or therapeutic targets based on the endogeneity of the gene set and the association with the phenotype. In this study, the candidate genes associated with glycolysis were obtained by WGCNA analysis.

### Construction of the Prognostic Signature

In this study, the glycolysis genes associated with prognosis were obtained initially by univariate Cox regression. Setting the domain value *p* < 0.05, the least absolute shrinkage and selection operator (LASSO) was performed, by which we can construct a penalty function and compress some regression coefficients to finally obtain the best prognostic signature. In this signature, a risk score can be calculated for each PC patient. Based on the median risk score value, the PC patients in the cohort could be divided into the high-risk and low-risk groups.

### Evaluation of the Prognostic Signature

Two independent external queues (PACA-AU and PACA-CA) were used to verify the accuracy of the model. The differences in prognosis, immune cells, and tumor mutation load between the high-risk and low-risk groups were compared, and the applicability of the model for different clinical characteristics was explored.

### Single-Cell Data Analysis

The “AUCell” package is an R package primarily used to quantify the level of enrichment of specific gene sets in each cell. In this study, a single-cell dataset of PC was analyzed to explore the activation of glycolytic pathways in different PC cell subtypes and to further assess the expression of genes in cell subtypes in the signature. The “monocle2” package is a mainstream R package for the analysis of single-cell mock cell trajectory differentiation. It was used to further analyze the epithelial cells in a proposed time series and to observe the changes of genes in the signature during this differentiation process.

### qRT-PCR to Verify the Expression of Seven Model Genes in PC

Next, the qRT-PCR experiment was performed on six PC patients, from whom the PC tissue and para-PC tissue were taken for mRNA quantification. These six patients were enrolled between June 2021 and October 2021 in Fuyang Hospital affiliated with Anhui Medical University. All of them signed informed consent forms. This study was approved by the Ethics Committee of the Fuyang Hospital affiliated with Anhui Medical University. The total cellular RNAs were isolated from cells using the TRIzol reagent (Invitrogen, Carlsbad, CA, United States), according to the manufacturer’s instructions. The reverse transcription was conducted using the reverse transcription kit provided by TaKaRa (Otsu, Shiga, Japan). Real-time polymerase chain reaction (RT-PCR) was performed using a QuantiTect SYBR Green PCR Kit (TaKaRa) and on an Applied Biosystems QuantStudio 1 system (Thermo, Waltham, MA, United States). Relative quantification was determined using the 2−^ΔΔCt^ method. The relative expression of messenger RNA (mRNA) for each gene was normalized to the level of glyceraldehyde-3-phosphate dehydrogenase (GAPDH) mRNA. The specific primer sequences adopted in this experiment are summarized in Supplementary Table S2.

## Results

### Prognostic Impact of Glycolysis on Pancreatic Cancer and Screening for Genes Associated With the Glycolytic Phenotype

**FIGURE 1:**
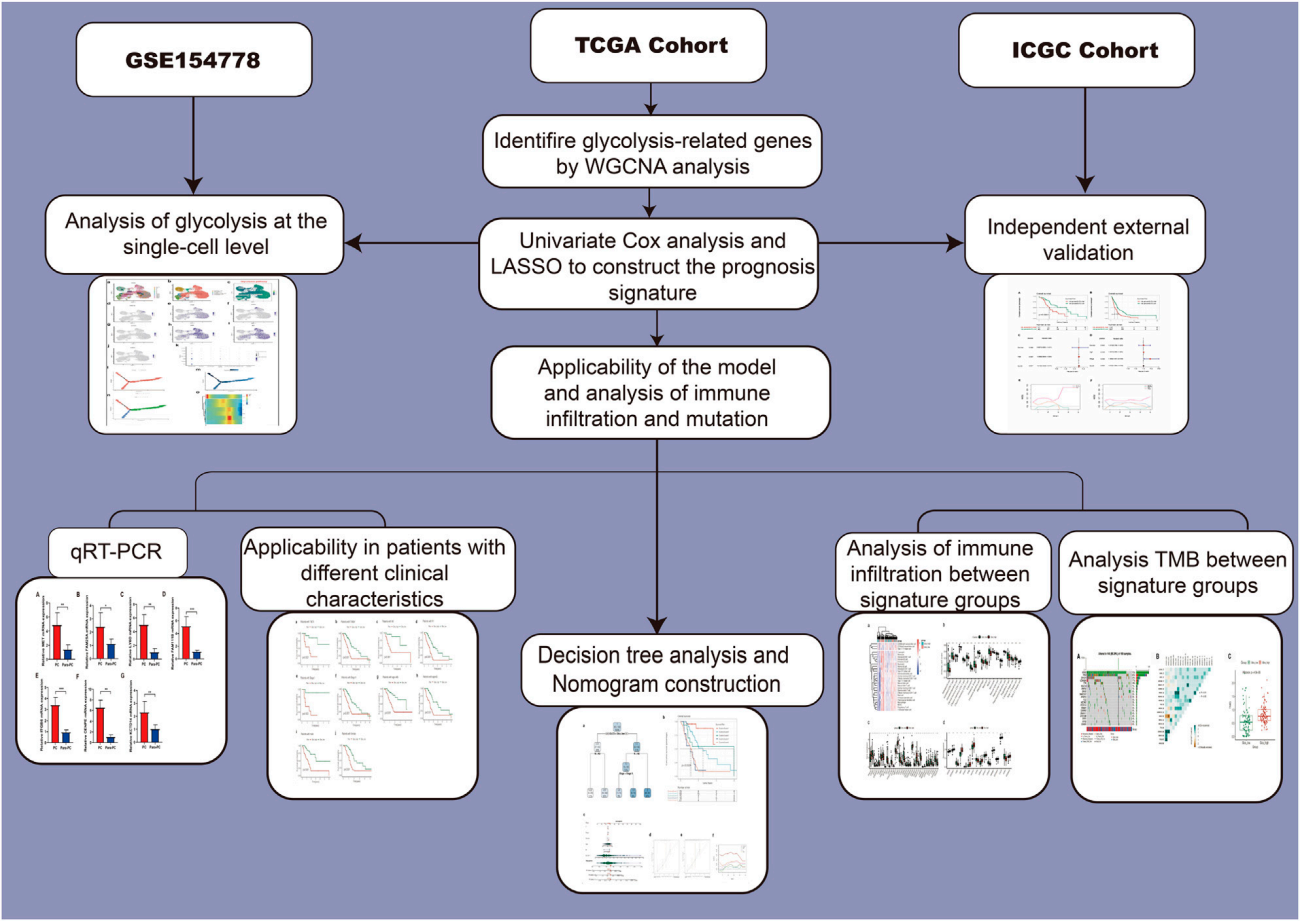
Flow chart of our study.

**FIGURE 2:**
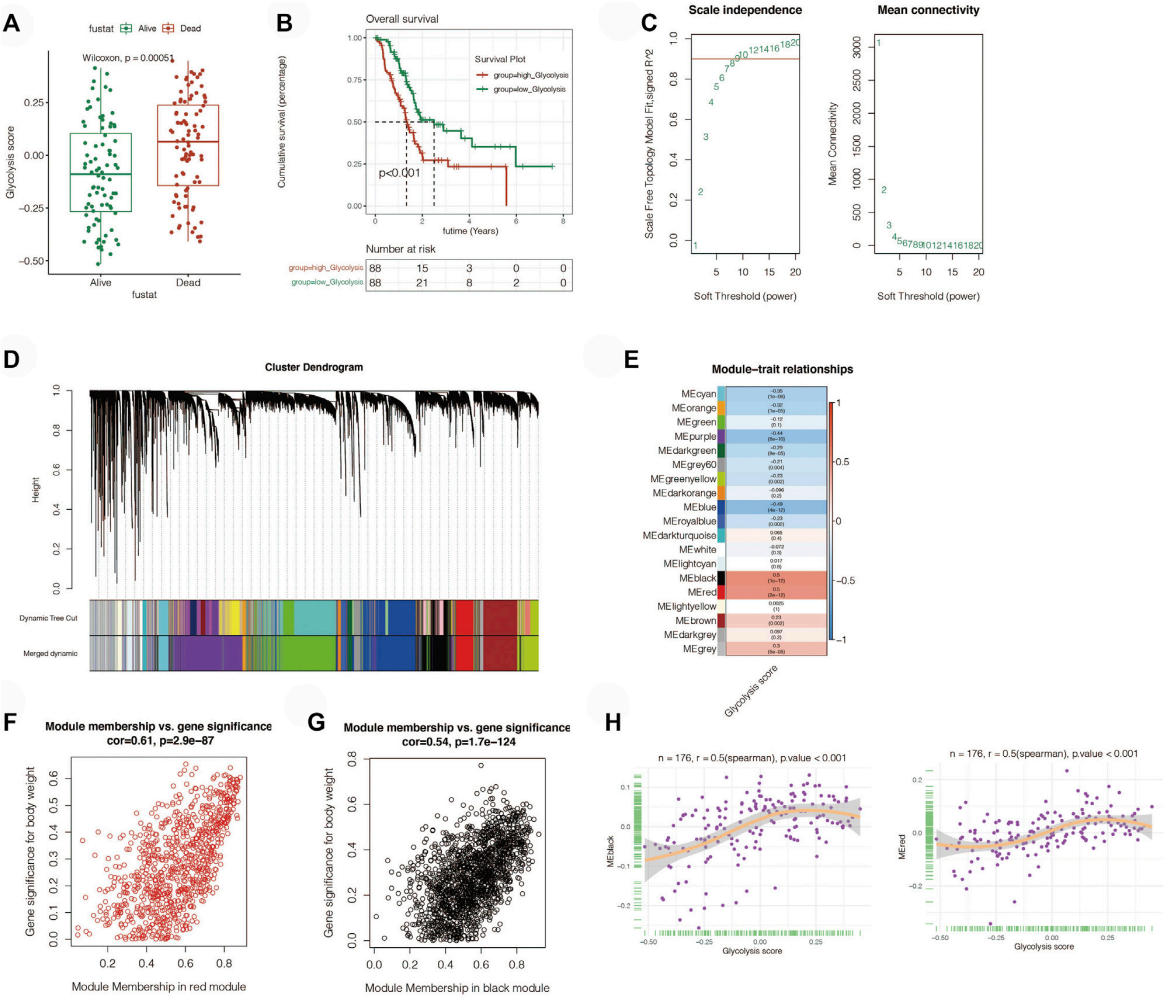
ssGSEA analysis and weighted gene correlation network analysis (WGCNA). **(A)** ssGSEA analysis showed that the glycolysis score was obvious elevated in the dead PC patients. **(B)** Survival analysis revealed that the high-glycolysis group has a worse prognosis. **(C)** Best soft threshold of WGCNA was 7. **(D)** WGCNA analysis found 18 no-gray gene modules. **(E)** Correlation between the modules and glycolysis. The black and red modules had the strongest correlation with the glycolytic phenotype (Cor = 0.5 and *p* < 0.001). **(F,G)** Relation between module membership and gene significance in red and black modules. **(H)** Correlation between red and black modules and glycolysis.

The main study flow of this study is shown in Figure 1. To compare the impact of glycolysis genes on patient prognosis in PC, in TCGA cohort, we quantified the glycolytic enrichment score of each PC patient using ssGSEA analysis and divided the patients into high- and low-glycolysis groups, according to the median value and found that the glycolytic enrichment score was higher in patients who died, and the prognosis of patients in the high-glycolysis group was poor (*p* < 0.001, Figures 2A,B). Moreover, in order to further search for genes associated with the glycolytic phenotype in PC, WGCNA analysis was performed. It was found that when the soft domain value was set to 7, R^2 > 0.8, suggesting that the data conformed to a power-law distribution and were suitable for subsequent analysis. The mean connectivity tended to be stable, suggesting that when the soft domain value was further increased, the effect on the results was not significant (Figure 2C). Subsequently, the minimum number of module genes was set to 100, deepSplit = 2, and the similar modules were merged by setting cutHight = 0.4, resulting in 18 non-gray gene modules, as shown in Figures 2D,E, among which we found that both black and red modules had the strongest correlation with the glycolytic phenotype (Cor = 0.5 & *p* < 0.001), suggesting that these two module genes are more closely related to glycolysis in pancreatic cancer. We also found a strong positive correlation between the module membership and gene importance in the red and black modules, as shown in Figures 2F,G (Cor = 0.61 & *p* < 0.001; Cor = 0.54 & *p* < 0.001). The correlation between the red module, black module, and glycolysis is shown in Figure 2H. We then selected the genes in the modules and set the p-value of the conditional GS to <0.0001 to obtain a total of 1,066 hub genes in the red and black modules, which were used in the subsequent one-way COX analysis.

### Construction of Glycolysis-Related Signatures

**FIGURE 3:**
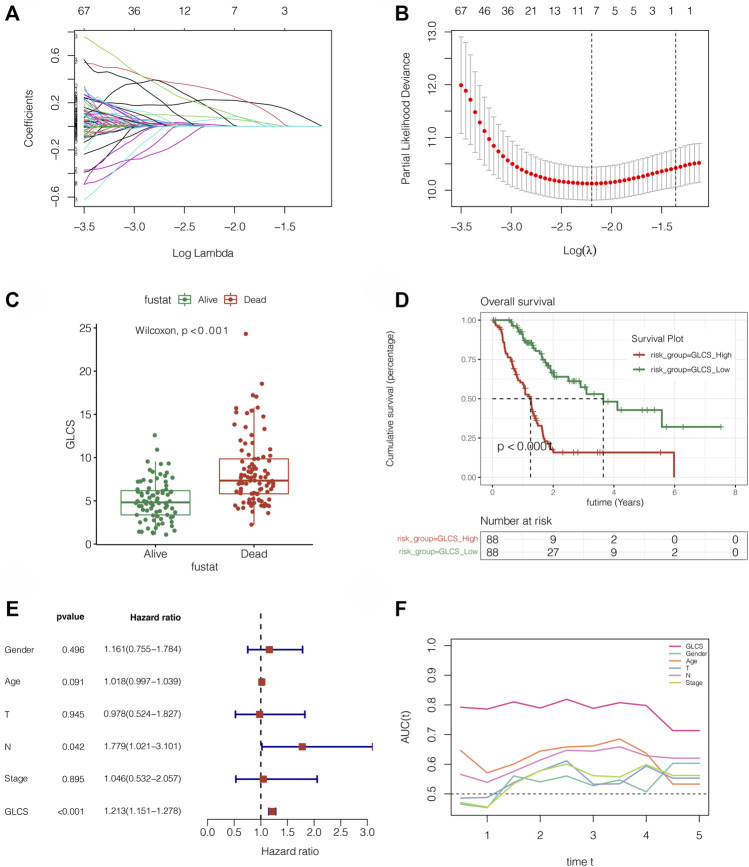
Gene signature was constructed in TCGA cohort. **(A,B)** LASSO Cox regression was used to identify the most important genes, and the optimal lambda was 0.111. **(C)** GLCS was obviously elevated in the dead PC patients (*p* = 2.3E-10). **(D)** Survival analysis reveals that GLCS-high has a worse prognosis (*p* < 0.0001). **(E)** Multivariate Cox analysis reveals that GLCS was an independent prognostic factor (*p* < 0.001). **(F)** AUC of GLCS and clinical features. The AUC value of GLCS was higher than that of other clinical features.

The aforementioned obtained hub genes were first initially screened by univariate Cox regression to get the genes related to prognosis. By setting *p* < 0.05, a total of 734 candidate genes were obtained. Next, Lasso regression was performed (Figures 3A,B). By setting the random seed to 55,555 and maxit = 1,000, the best lambda value is obtained as 0.111., Finally, we got the signature made up of seven genes (*MET*, *FAM25A*, *LY6D*, *FAM111B*, *ITGB6*, *CENPE*, and *KCTD14*). The signature value was calculated by the following formula: GLCS = MET*0.224 +FAM25A*0.306 +LY6D*0.076 +FAM111B*0.060 +ITGB6 *0.012 +CENPE*0.128 +KCTD14*0.149. All PC samples were divided into the GLCS high-risk group and GLCS low-risk group, according to the median value of the signature (GLCS). The prognosis of patients between the different subgroups of the signature was subsequently compared, as shown in Figures 3C,D. The GLCS score was different between dead and alive patients, and the GLCS score was higher in dead patients. (*p* < 0.001). The survival curve analysis suggested that the prognosis of patients in the GLCS-high group was worse (*p* < 0.001). After multivariate Cox analysis, it was found (as in Figure 3E) that GLCS was an independent prognostic influence compared to other clinical characteristics (*p* < 0.001). Subsequently, the sequential ROC curve analysis (Figure 3F) revealed that the area under the curve (AUC) of GLCS for the assessment of prognosis of pancreatic cancer patients was around 0.8, which was superior to other clinical characteristics, such as gender, age, and tumor stage. In addition, we also analyzed the correlation between the seven model genes and the glycolysis phenotype, and the results are shown in Supplementary Figure S1.

### Validation of This Signature Accuracy in Two Independent External Sets

**FIGURE 4:**
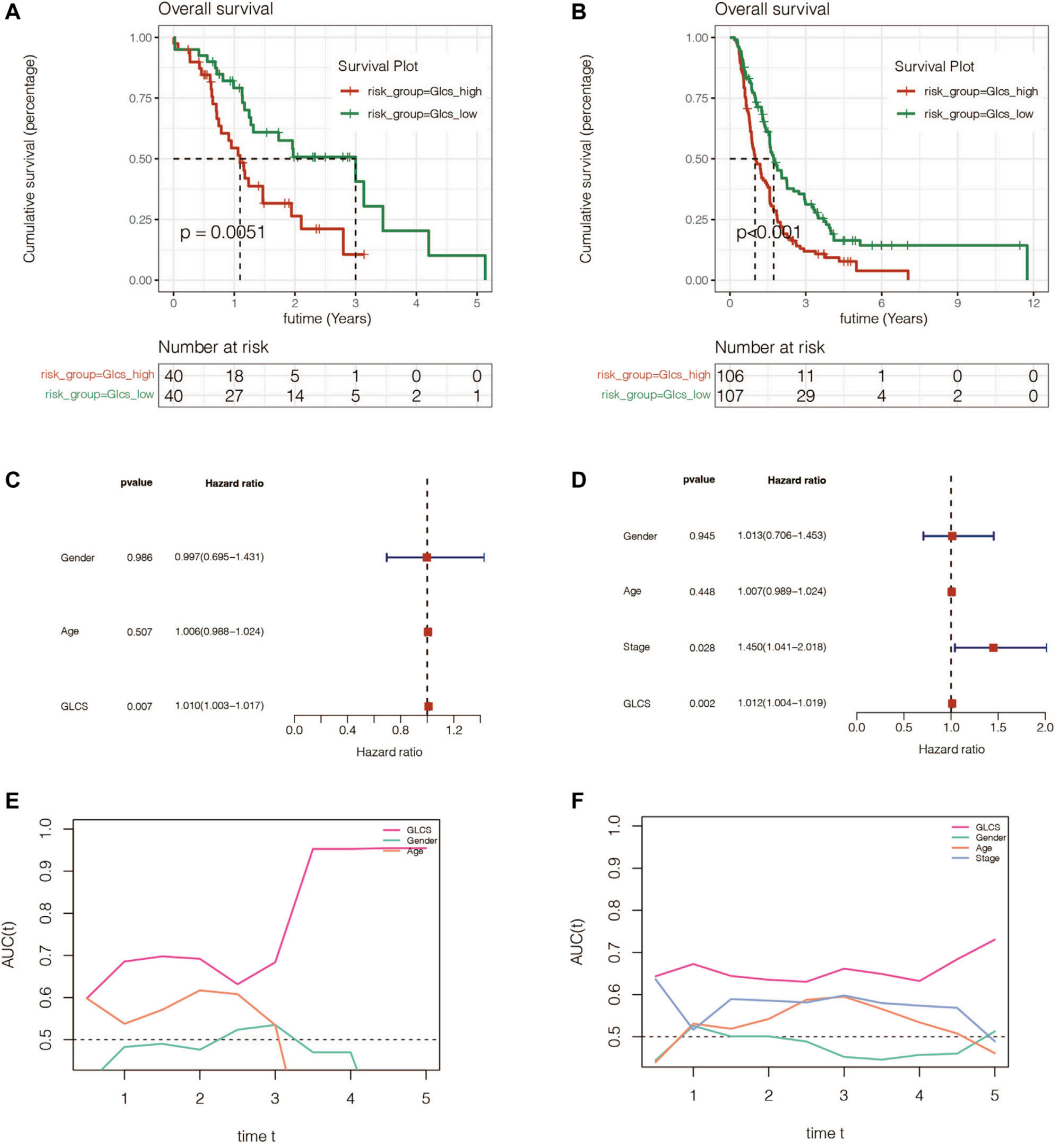
Assessment of the gene signature in extra validation cohorts. **(A)** Survival analysis in the PACA-AU cohort suggested that the prognosis of the GLCS-high group was worse (*p* = 0.0051). **(B)** Survival analysis in the PACA-CA cohort suggested that the prognosis of the GLCS-high group was worse (*p* < 0.001). **(C,D)** Multivariate Cox analysis in the PACA-AU cohort and PACA-CA cohort revealed that GLCS was an independent prognostic factor. **(E,F)** AUC of GLCS and clinical features in the PACA-AU cohort and PACA-CA cohort.

To further validate the stability and accuracy of the signature, the PACA-AU and PACA-CA cohorts were used for independent external validation. As shown in Figures 4A,B, the survival curve analysis suggested that the prognosis of the GLCS-high group was worse in both external validation sets, with *p* = 0.0051 in the PACA-AU cohort and *p* < 0.001 in the PACA-CA cohort. To further verify whether GLCS could be used as an independent prognostic influence, as shown in Figures 4C,D, it was found that in the PACA-AU cohort, only GLCS was an independent prognostic influencing factor, while both GLCS and Stage were independent prognostic influencing factors in the PACA-CA cohort. As shown in Figures 4E,F, the continuous ROC analysis over time in the two external validation sets found that the AUC value of GLCS was maintained at around 0.7 and superior to other clinical indicators. In conclusion, GLCS was an independent prognostic influencing factor in both PACA-AU and PACA-CA cohorts, patients in the GLCS-high group had a poorer prognosis, and the prognostic diagnostic value of GLCS for PC patients was superior to that of the other clinical indicators.

### Signature Performed Well in PC With Different Clinical Characteristics

**FIGURE 5:**
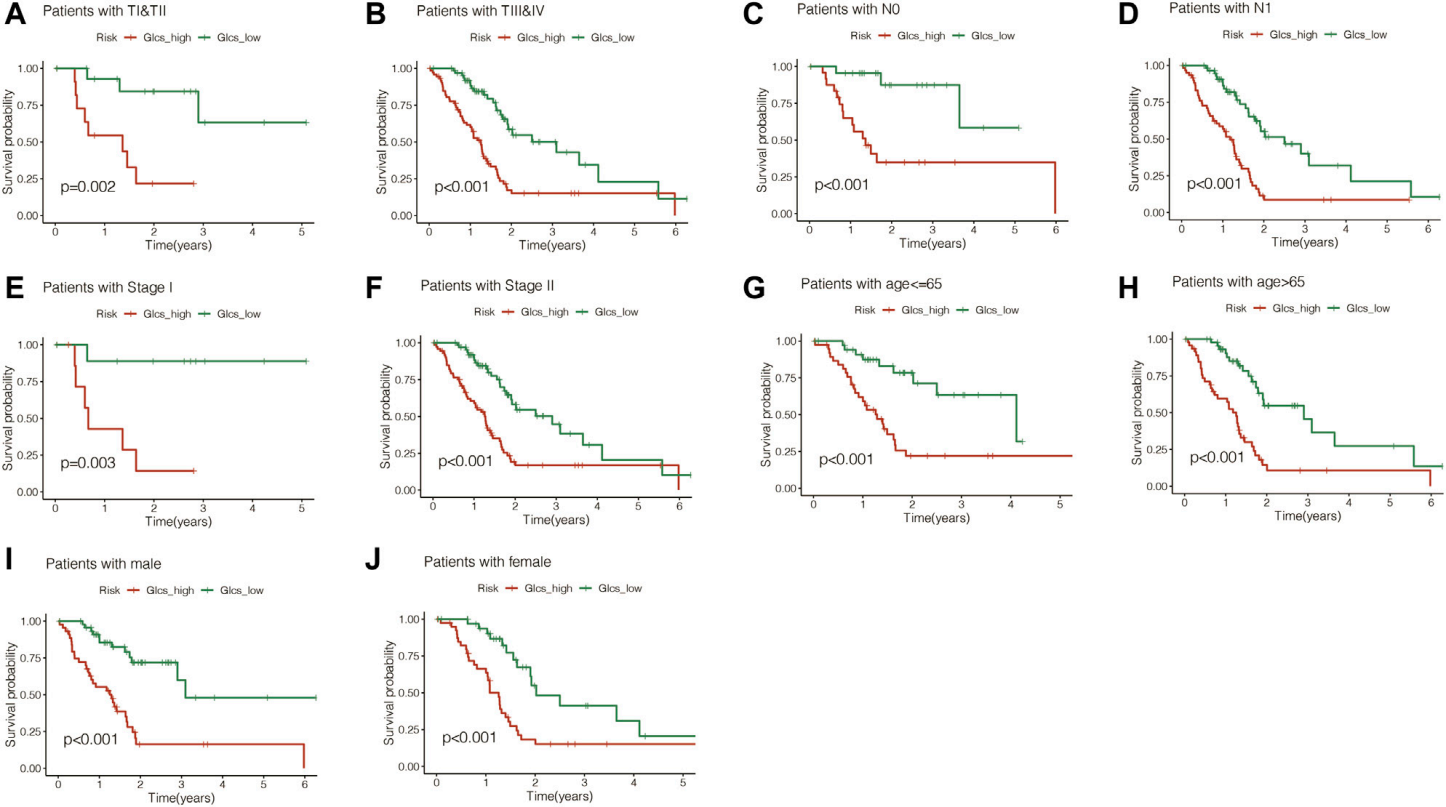
Gene signature is suitable for different clinical patients. **(A)** Among the TI and TII stage PC patients, the prognosis of the high-GLCS group was worse (*p* = 0.002). **(B)** Among the TIII&TIV stage PC patients, the prognosis of the high-GLCS group was worse (*p* < 0.001). **(C)** Among the PC patients with N0, the prognosis of the high-GLCS group was worse (*p* < 0.001). **(D)** Among the PC patients with N1, the prognosis of the high-GLCS group was worse (*p* < 0.001). **(E)** Among PC patients with stage I, the prognosis of the high-GLCS group was worse (*p* = 0.003). **(F)** Among PC patients with stage II, the prognosis of the high-GLCS group was worse (*p* < 0.001). **(G)** Among PC patients with age<=65, the prognosis of the high-GLCS group was worse (*p* < 0.001). **(H)** Among PC patients with age>65, the prognosis of the high-GLCS group was worse (*p* < 0.001). **(I)** Among male PC patients, the prognosis of the high-GLCS group was worse (*p* < 0.001) **(J)** Among female PC patients, the prognosis of the high-GLCS group was worse (*p* < 0.001).

To investigate whether the signature is equally valid in PC patients with different clinical characteristics, the patients were grouped according to different clinical characteristics in TCGA cohort. It was found that pancreatic cancer patients in the GLCS-high group were always associated with significantly worse prognosis, whether grouped by T-stage, N-stage, total stage, age, and gender, suggesting that the signature remains applicable in a population with different clinical characteristics (Figures 5A–J).

### Exploring the Differences in Immune Infiltration Between GLCS-High and GLCS-Low Groups

**FIGURE 6:**
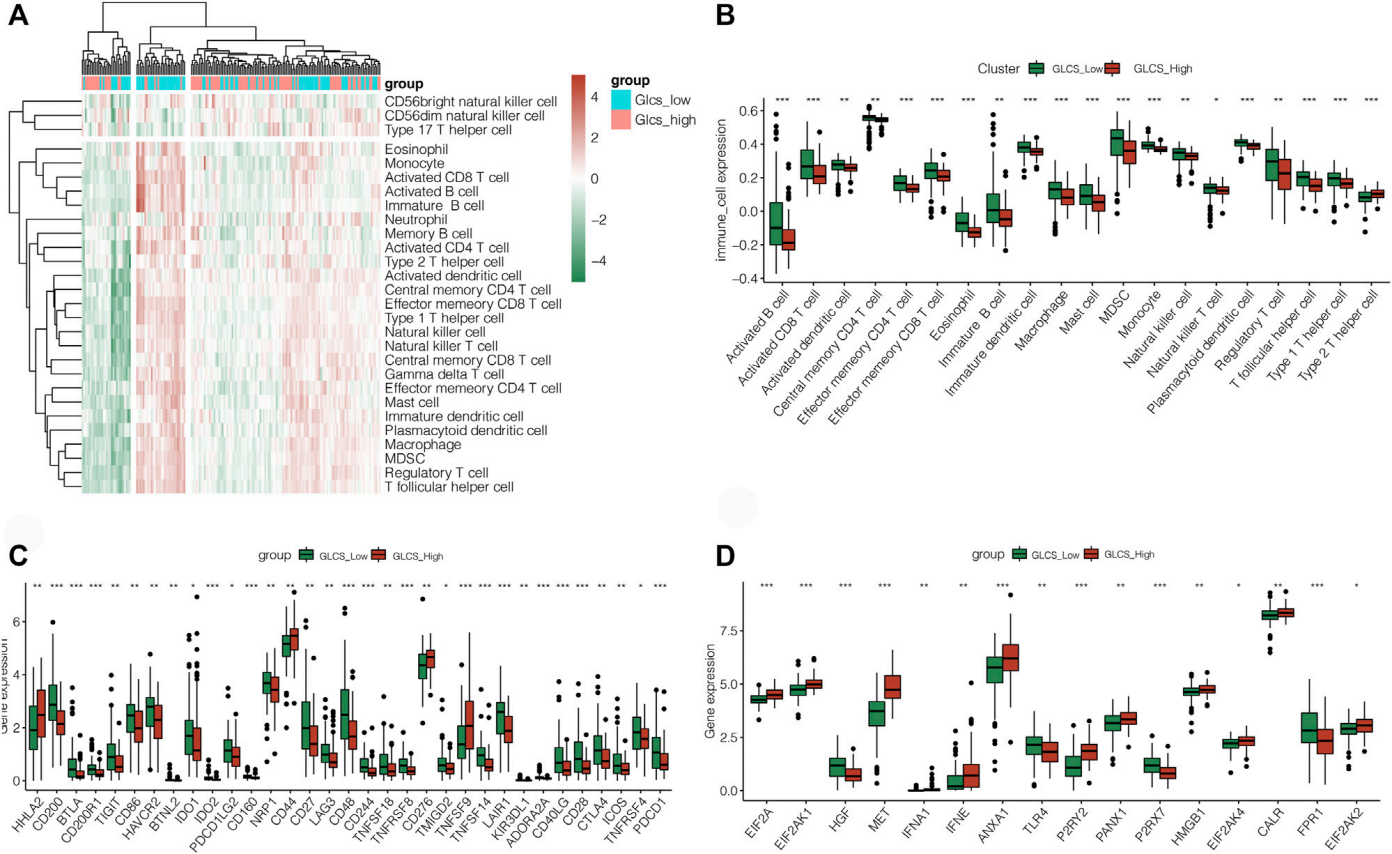
Exploration of the relation between GLCS and immune infiltration. **(A)** Immune landscape of PC patients. **(B)** Difference in immune infiltration levels between the two groups in the form of a box plot. **(C)** Differences in the immune checkpoint gene expression between high-risk and low-risk groups. **(D)** Differences in the expression of immunogenic cell death genes between high-risk and low-risk groups (*p < 0.05, **p < 0.01, and ***p < 0.001).

The previous results found that patients in the GLCS-high group had a poorer prognosis. Then, we further investigated the differences in immune cells and immune check points (ICPs) and immunogenic cell death (ICDs) between the GLCS-high and GLCS-Low groups. Figure 6A shows the immune landscape between the GLCS-high group and the GLCs-Low group, and Figure 6B shows the difference in immune infiltration levels between the two groups in the form of a box plot. From there, we can see that the trend of immune infiltration levels in the GLCs-high group is lower, which may be related to its worse prognosis. It was found that 33 immune checkpoint genes were differentially expressed between the GLCS-high and GLCS-Low groups (Figure 6C). Only four immune checkpoint genes, *HHLA2*, *CD44*, *CD276*, and *TNFSF9*, were highly expressed in the GLCS-high group, and 29 immune checkpoint genes were highly expressed in the GLCS-Low group, such as *PDCD1*, *CTLA4*, *PDCD1LG2*, and *CD86* (Figure 6C).

Interestingly, we found that immunogenic cell death (ICDs) genes were differentially expressed in the GLCS-high and GLCS-Low groups (Figure 6D). *EIF2A*, *EIF2AK1*, *MET*, *IFNA1*, *IFNE*, *ANXA1*, *P2RY2*, *PANX1*, *HMGB1*, *EIF2AK4*, *CALR*, and *EIF2AK2* were highly expressed in the GLCS-high group, and *HGF*, *TLR4*, *P2RX7*, and *FPR1* were highly expressed in the GLCS-Low group. In conclusion, the immune cells were less enriched in the GLCS-high group, immune checkpoint genes were less expressed in the GLCS-high group, and immunogenic cell death (ICD) genes were highly expressed mainly in the GLCS-high group, which might be a factor contributing to the poorer prognosis of patients in the GLCS-high group.

### Exploring the Mutational Landscape Between GLCS-High and GLCS-Low Groups

**FIGURE 7:**
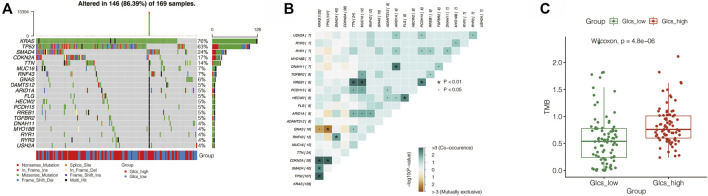
Exploration of the relation between GLCS and tumor mutation. **(A)** Landscape of genetic mutations in PC patients. **(B)** Mutation symbiosis among the top mutation genes. **(C)** TMB were higher in the GLCS-high group.

Gene mutations are an important influential factor in the prognosis of tumor patients. To investigate the mutation of genes in the GLCS-high and GLCS-Low groups in PC patients, the “maftools” R package was used to map the mutation landscape of PC patients. It was found that the top 20 genes with the highest mutation frequency were mutated in 86.39% of patients, and the top two most mutation-prone genes were *KRAS* and *TP53* (Figure 7A). Both mutation types were dominated by missense_mutation, and they were mainly distributed in the GLCS-high group. Moreover, we analyzed the mutation symbiosis of the top 20 genes and found that mutation symbiosis existed between KRAS and GNAS (*p* < 0.05), between KRAS and CDKN2A, SMAD4, TP53 (*p* < 0.05), and between TP53 and GNAS and CDKN2A (*p* < 0.01) (Figure 7B). As shown in Figure 7C, the distribution of the mutation number and tumor mutational load (TMB) was different in the two groups, and the number of mutations and TMB were higher in the GLCS-high group (*p* < 0.001).

### Probing Glycolytic Pathway Activation in PC Cell Subtypes

**FIGURE 8:**
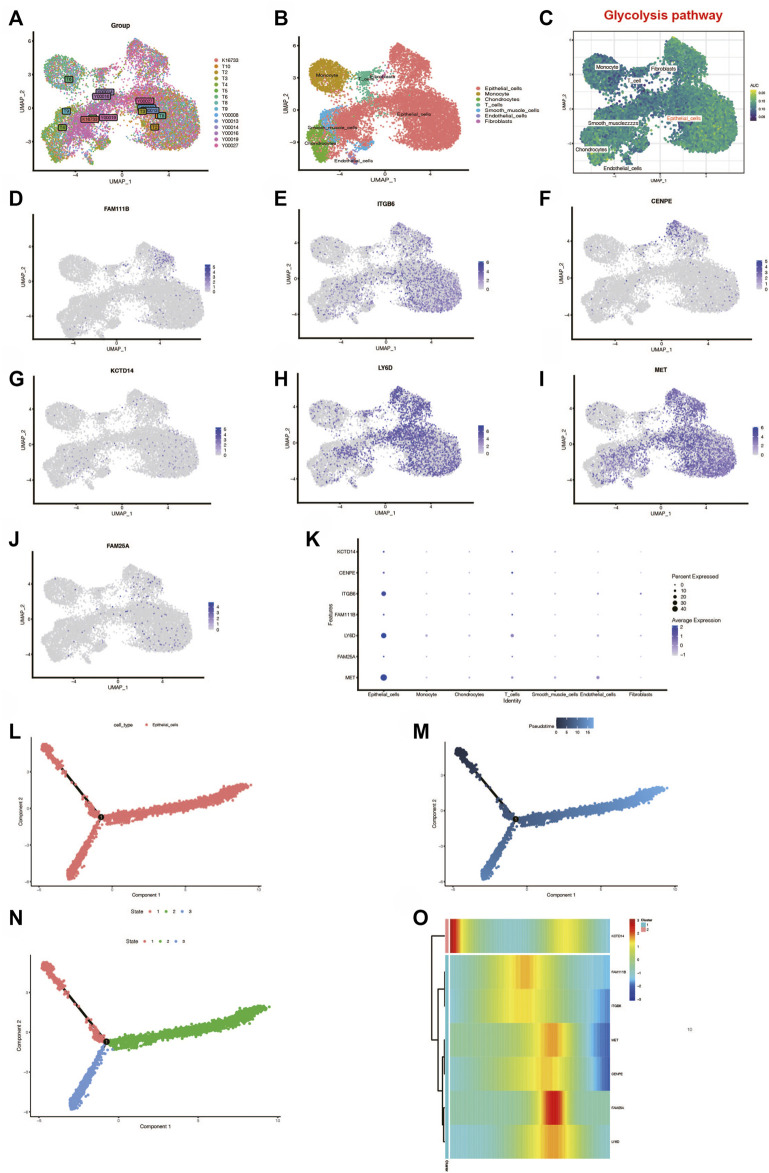
Single-cell analysis. **(A)** Fifteen samples were integrated with the CCA method. **(B)** Dimension reduction and cluster analysis. The cell types were shown with the umap plot. **(C)** Glycolysis pathway was activated in different cell types **(D–K)**. Genes in the prognostic signature expressed differently in different cell types. **(L–N)** Analysis of epithelial cell locus differentiation and **(O)** the genes in the signature expressed differently during the epithelial cell locus differentiation.

To further investigate the activation of the glycolytic pathway in pancreatic cancer cell subtypes, we performed a subsequent analysis of single-cell sequencing samples from PC. Also, the 15 PC samples were first integrated by the “CCA” method, as shown in Figure 8A, and we found that these samples were more uniformly distributed without significant batch effects and suitable for subsequent analysis. Subsequently, we obtained a total of 16 cell clusters by principal component analysis with reduced dimensionality. The cells were annotated with the SingleR package and could be roughly annotated as seven cell subtypes: epithelial cells, monocytes, chondrocytes, T cells, slippery muscle cells, endothelial cells, and fibroblasts (Figure 8B). Then, to further investigate the enrichment of the glycolytic pathway in different cell types, we performed the scoring of the pathway among various cell types using the AUCell package and found that the glycolytic pathway has higher AUC values in epithelial cells, suggesting that the glycolytic pathway is more enriched in this cell type (Figure 8C). Interestingly, we found that seven genes in the signature *FAM111B*, *CENPE*, *KCTD14*, *FAM25A*, *MET*, *LY6D*, and *ITGB6* were all expressed mainly in the epithelial cells, especially *ITGB6*, *LY6D*, and *MET* (Figures 8D–K). In order to further investigate the relationship between epithelial cell development and genes in the signature, we selected all epithelial cells and visualized the results through the “monocle2” package. By setting the method as “DDRtree” and max_components as 2, it was found that the epithelial cell differentiation process produced two branches, and the darker blue color in the upper left suggests that differentiation occurs earlier, from deeper blue to lighter blue (Figures 8I–M). Interestingly, we found that there are three differentiation states during epithelial cell differentiation (Figure 8N). State 1 in the upper left is the earlier differentiation state. State 2 and State 3 are later differentiation. We also found that the expression of one gene in the signature, KCTD14, showed a decreasing state during the differentiation of epithelial cells. In contrast, the expression of the remaining six genes showed an up and then down.

### Clinical Implications of the Signature

**FIGURE 9:**
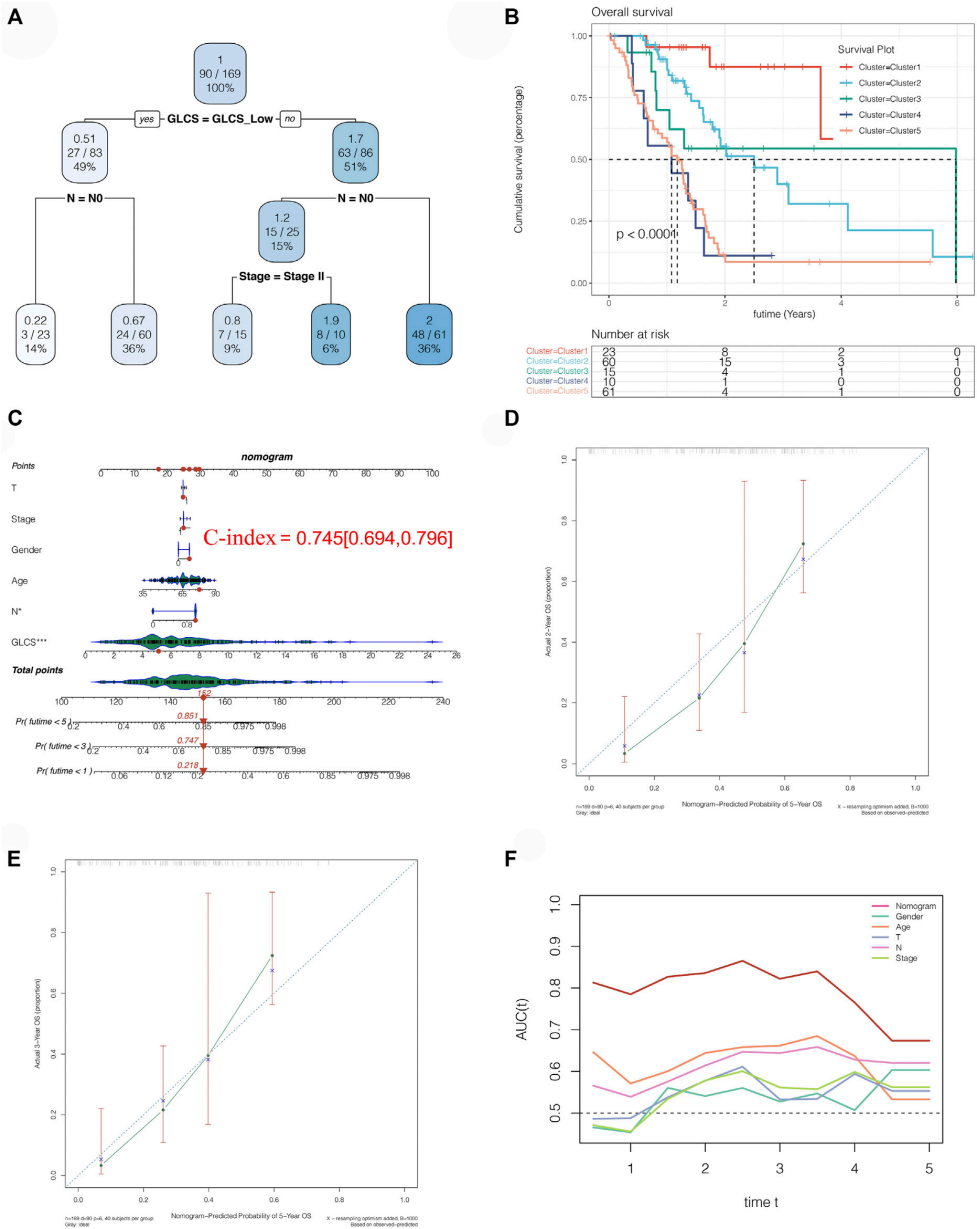
Clinical implications of the signature. **(A)** Decision tree analysis could divide PC patients into five risk subtypes. **(B)** Five risk subtypes have different prognosis, and cluster 1 has a best prognosis. **(C)** Nomogram analysis showed the 1-, 3-, and 5-year mortality rates of patient TCGA-S4-A8RP. **(D,E)** 2- and 3-year calibration curves of the nomogram. **(F)** AUC of the nomogram and other clinical features to evaluate the prognosis of PC.

We performed a decision tree analysis of PC patients using the “rpart” R package. It was found that patients could be divided into five groups based on the high and low expressions of GLCS, N0 stage, and Stage II stage. Through survival analysis, we found that there were differences in prognosis among these five groups (Figure 9A). Among these five groups, cluster 1 had the best prognosis but cluster 4 had the worst prognosis (Figure 9B). To further guide the clinic, we constructed a nomogram, as shown in Figure 9C. By comparing the GLSC values of the patient with the clinical characteristics, we could predict the 1-, 3-, and 5-year mortality rates of 21.8, 74.7, and 85.1% for this patient, which could help guide some clinical decisions and treatment options. Moreover, we found that the accuracy of the prognosis at 2 and 3 years predicted by this nomogram is also relatively high, as shown in Figures 9D,E. Furthermore, the AUC value of the nomogram for predicting the prognosis of patients over time is around 0.8, which is better than other clinical indicators (Figure 9F). The glycolysis score in different clusters and in the high-risk and low-risk groups of the aforementioned three cohorts is shown in Supplementary Figure S2.

### qRT-PCR to Verify the Expression of Seven Model Genes in PC

**FIGURE 10:**
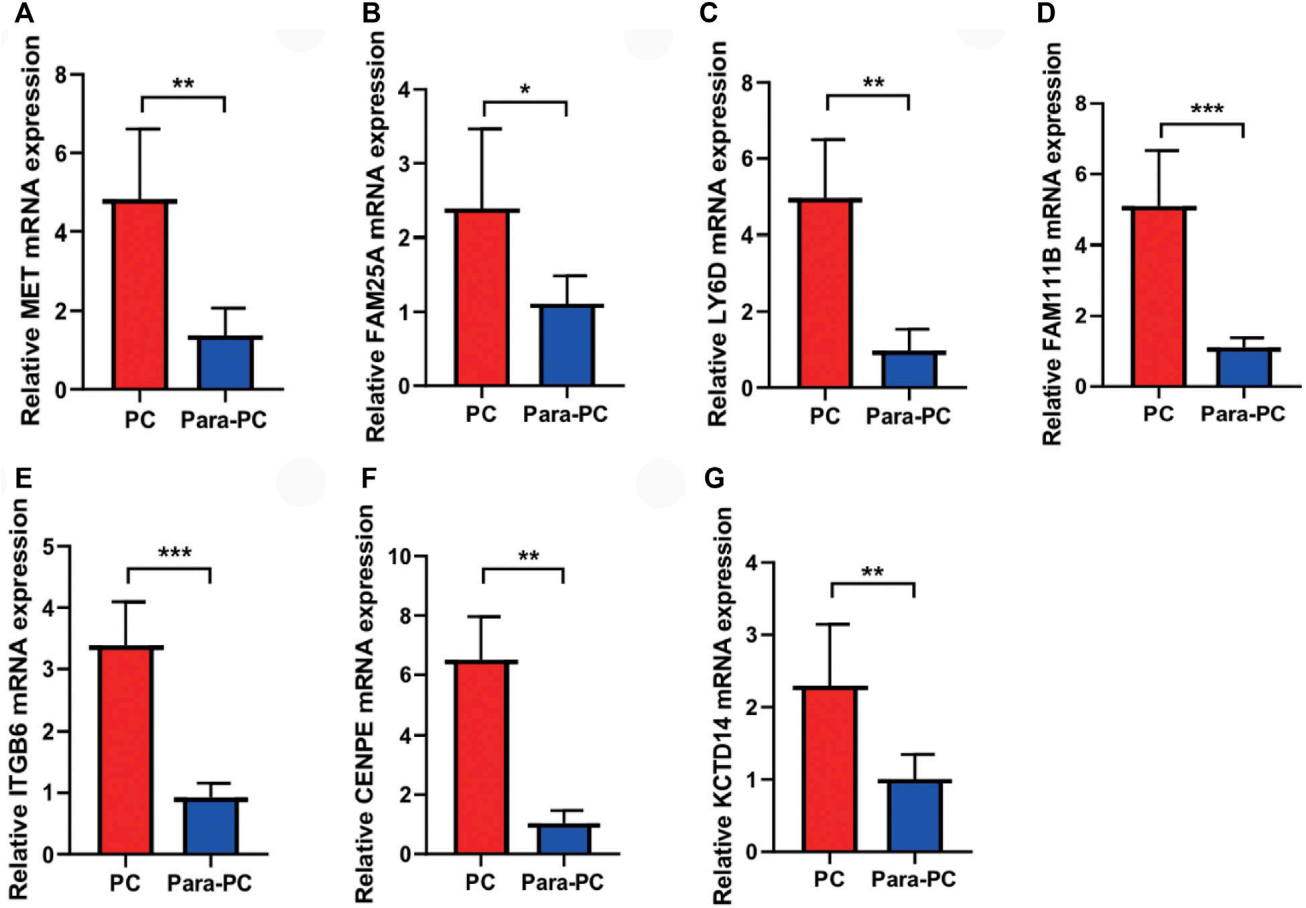
qRT-PCR to verify the expressions of seven model genes in PC. The seven model genes were all upregulated in PC compared with the normal adjacent tissues (*p < 0.05, **p < 0.01, and ***p < 0.001). **(A)** MET; **(B)** FAM25A; **(C)** LY6D; **(D)** FAM111B; **(E)** ITGB6; **(F)** CENPE; **(G)** KCTD14.

Next, we used qRT-PCR to detect the expression of seven model genes in PC. The results showed that seven model genes were all upregulated in PC compared with normal adjacent tissues (*p < 0.05, **p < 0.01, and ***p < 0.001; Figure 10). In addition, we used the HPA database to verify the seven model genes at the protein level, and the results are shown in Supplementary Figure S3.

## Discussion

There has been a renewed interest in tumor glycolysis in recent years (Cascone et al., 2018). Increased glucose uptake and glycolysis are characteristic of cancer and can contribute to tumor progression by accelerating the growth of tumor cells and thus tumor progression (Li et al., 2016; Fang et al., 2020). Aberrant cancer cell metabolism has been shown to play an important role in tumor progression and is a hot topic of research for investigators (Fang et al., 2020). Recently, metabolic reorganization has been found to be one of the new features of cancer that may be associated with patient prognosis (Gong et al., 2016). Moreover, among the different types of metabolic reorganization, accelerated aerobic glycolysis is an important phenotype of metabolic reorganization in cancer (Cascone et al., 2018). Through aerobic glycolysis, it can provide the molecules required for cancer cell growth and proliferation for new cells and play an important role in maintaining cellular redox during proliferation. (Cascone et al., 2018) Studies on glycolysis of various tumors have found that oncogenic pathways promote tumorigenesis and development by regulating tumor glycolysis, especially in proliferation and angiogenesis (Shang et al., 2019; Li et al., 2020a), such as in liver cancer and breast cancer. However, in pancreatic cancer, few people have studied and developed a glycolysis-related gene signature to predict the patient prognosis and assess the patient risk for individualized management of clinical patients.

In our study, we found that glycolysis is a prognostic risk factor in PC, which is consistent with previous evidence that glycolytic pathways promote tumor progression and are associated with poor patient prognosis. We then searched for a set of genes most associated with glycolysis by WGCNA and then constructed a prognostic signature; to validate the stability and the accuracy of the signature, we validated the signature in two additional independent external data cohorts and found that the gene signature was an independent prognostic influencer in pancreatic cancer patients and could better distinguish the high-risk patients, and we also validated the signature by. We also found that the gene signature was more accurate than the clinical TNM system and gender-age in the prognostic assessment of patients by continuous-time prognostic ROC analysis. Furthermore, we found the differences in multiple immune cells and mutations between the two subgroups of the gene signature and that activated CD8 T cells, central memory CD4 T cells, effector memory CD4 cells, and effect memory CD8 T cells were infiltrated more in the GLCS-Low group than in the GLCS-high group. It has been found that activated T cells can inhibit the glycolytic pathway and thus inhibit the progression of PC (Cascone et al., 2018), which is consistent with the results in our study that more activated T cells in the GLCS-Low group had a better prognosis for patients. It was found that tumor mutational burden (TMB) is identified as a biomarker for response to immunotherapy in several cancer types and is often associated with poor prognosis (Li et al., 2020b), and in this study, we found that the value of TMB in the GLCS-Low group was lower than that in the GLCS-high group and that the prognosis was worse in the GLCS-high group. Subsequently, our analysis of the dataset of pancreatic cancer single-cell sequencing revealed that the glycolytic pathway was mainly activated in epithelial cells, and seven genes in the gene signature were also mainly expressed in epithelial cells, especially *ITGB6*, *LY6D*, and *MET*, suggesting that epithelial cells may play an important role in the progression of PC. We further investigated the cell differentiation trajectory of epithelial cells and found that there were two branches of epithelial cells, and the expressions of *ITGB6*, *LY6D*, and *MET* in the gene signature mainly showed an increasing and then decreasing trend during development, while interestingly, the expression of KCTD14 mainly showed a decreasing, then increasing, and then decreasing trend, which may suggest that the genes in this gene signature may have a role in epithelial cell development. Finally, in order to facilitate the risk subgroup classification and personalized management of clinical patients, we performed decision tree analysis and constructed a nomogram to classify the PC patients into five risk subgroups based on the risk values of the gene signature and clinical characteristics and combined with the nomogram to evaluate the prognosis of patients to facilitate personalized management of clinical patients.

Currently, the studies have elucidated the significance of seven genes in this signature in pancreatic diseases. The MET gene plays an important role in the proliferation and progression of pancreatic cancer through the hepatocyte growth factor (HGF)/C-MET axis (Wang et al., 2020). A clinical study conducted by Lux et al. (2019) found that a high serum MET expression was a poor prognostic indicator in patients with pancreatic cancer. The role of FAM25A in pancreatic cancer is still unclear. Kalloger et al. (2021)found that the upregulation of the LY6D expression was associated with poor prognosis in patients with pancreatic cancer. Seo et al. (2016) found that FAM111B was associated with autosomal dominant exocrine pancreatic dysfunction. Lenggenhager et al. (2021)found that ITGB6 is a potential early biomarker of pancreatic cancer, which can improve the accuracy of early diagnosis of pancreatic cancer. Zhuang et al. (2020) performed the bioinformatic analysis and found that ITGB6 is a poor prognostic indicator of pancreatic cancer and is associated with Notch pathway activation and immune suppression. Mayes et al. (2013)found that inhibition of CENPE inhibited the growth activity of pancreatic cancer cells. Piccolo et al. (2021)found that KCTD14 was associated with type 2 diabetes in mice and was involved in mediating the regulation of the nutritional environment in the digestive tract. In our study, the prognostic signature constructed by these seven genes can not only guide the prognosis of patients with pancreatic cancer but also provide a reference for the exploration of the immune microenvironment of pancreatic cancer (Liu et al., 2021).

The reasons for the poor prognosis of pancreatic cancer include delayed diagnosis, lack of early specific serological markers, invasive growth, early metastasis, and resistance to chemotherapy/radiotherapy (Goral, 2015). At the same time, pancreatic cancer is associated with considerable immune escape (Morrison et al., 2018). The immune escape in pancreatic cancer is characterized by an immunosuppressive microenvironment and less immunogenicity due to low mutation load (Schizas et al., 2020). This is one reason why immunotherapies, such as immune checkpoint blockade, do not work well in pancreatic cancer (Schizas et al., 2020). Currently, the conventional immunotherapy regimens have only been approved for pancreatic cancer patients with microsatellite instability and mismatch repair defects (Schizas et al., 2020). Multiple combination therapies are being developed (Wu et al., 2021). Our study provides an immunological landscape of pancreatic cancer, from which we can visually observe differences in levels of immune cell infiltration between high-risk and low-risk groups. In addition, we also explored the expression of immune checkpoint-related genes and immunogenic cell death genes in the two groups. This deepens our understanding of the immune microenvironment of pancreatic cancer and provides a reference for immunotherapy of pancreatic cancer.

In general, our study comprehensively analyzed single-cell sequencing data and transcriptome data and thus constructed the glycolysis-related gene prognostic signature, which has certain significance in guiding the prognosis and immunotherapy of pancreatic cancer patients. But there are limitations to our study. We only conducted the PCR experiments to detect the expression of seven genes of this signature in pancreatic cancer and normal tissues and lacked further functional experiments to verify the function of the genes, which we will make improvements in the future.

## Conclusion

In conclusion, we found that glycolysis is an influential factor in the prognosis of PC. Furthermore, we constructed a glycolysis-related gene tag to assess the prognosis of PC patients and validated the tag in several external independent cohorts and found that the tag performed well and had high stability. The glycolytic pathway may be more activated in the epithelial cells of pancreatic cancer. The decision trees and nomograms facilitate personalized clinical management of PC patients.

## Data Availability

The original contributions presented in the study are included in the article/Supplementary Material; further inquiries can be directed to the corresponding authors.

## References

[B1] Abel E. V. Simeone D. M. (2013). Biology and Clinical Applications of Pancreatic Cancer Stem Cells. Gastroenterology 144 (6), 1241–1248. 10.1053/j.gastro.2013.01.072 23622133

[B2] Allenson K. Castillo J. San Lucas F. A. Scelo G. Kim D. U. Bernard V. (2017). High Prevalence of mutantKRAS in Circulating Exosome-Derived DNA from Early-Stage Pancreatic Cancer Patients. Ann. Oncol. 28 (4), 741–747. 10.1093/annonc/mdx004 28104621 PMC5834026

[B3] Cascone T. McKenzie J. A. Mbofung R. M. Punt S. Wang Z. Xu C. (2018). Increased Tumor Glycolysis Characterizes Immune Resistance to Adoptive T Cell Therapy. Cell Metab. 27 (5), 977–987. e4. 10.1016/j.cmet.2018.02.024 29628419 PMC5932208

[B4] Duan B. Hu J. Liu H. Wang Y. Li H. Liu S. (2018). Genetic Variants in the Platelet-Derived Growth Factor Subunit B Gene Associated with Pancreatic Cancer Risk. Int. J. Cancer 142 (7), 1322–1331. 10.1002/ijc.31171 29168174 PMC5805574

[B5] Eissa M. A. L. Lerner L. Abdelfatah E. Shankar N. Canner J. K. Hasan N. M. (2019). Promoter Methylation of ADAMTS1 and BNC1 as Potential Biomarkers for Early Detection of Pancreatic Cancer in Blood. Clin. Epigenet 11 (1), 59. Published 2019 Apr 5. 10.1186/s13148-019-0650-0 PMC6451253 30953539

[B6] Fang E. Wang X. Wang J. Hu A. Song H. Yang F. (2020). Therapeutic Targeting of YY1/MZF1 axis by MZF1-uPEP Inhibits Aerobic Glycolysis and Neuroblastoma Progression. Theranostics 10 (4), 1555–1571. Published 2020 Jan 1. 10.7150/thno.37383 32042322 PMC6993229

[B7] Gong Y. Ma Y. Sinyuk M. Loganathan S. Thompson R. C. Sarkaria J. N. (2016). Insulin-mediated Signaling Promotes Proliferation and Survival of Glioblastoma through Akt Activation. Neuro Oncol. 18 (1), 48–57. 10.1093/neuonc/nov096 26136493 PMC4677408

[B8] Goral V. (2015). Pancreatic Cancer: Pathogenesis and Diagnosis. Asian Pac. J. Cancer Prev. 16 (14), 5619–5624. 10.7314/apjcp.2015.16.14.5619 26320426

[B9] Guo T. Liu D. Peng S. Wang M. Li Y. (2021). A Positive Feedback Loop of lncRNA MIR31HG-miR-361-3p -YY1 Accelerates Colorectal Cancer Progression through Modulating Proliferation, Angiogenesis, and Glycolysis. Front. Oncol. 11, 684984. Published 2021 Aug 17. 10.3389/fonc.2021.684984 34485123 PMC8416113

[B10] Kalloger S. E. Karasinska J. M. Keung M. S. Thompson D. L. Ho J. Chow C. (2021). Stroma vs Epithelium‐enhanced Prognostics through Histologic Stratification in Pancreatic Ductal Adenocarcinoma. Int. J. Cancer 148 (2), 481–491. 10.1002/ijc.33304 32955725

[B11] Lenggenhager D. Bengs S. Fritsch R. Hussung S. Busenhart P. Endhardt K. (2021). β6-Integrin Serves as a Potential Serum Marker for Diagnosis and Prognosis of Pancreatic Adenocarcinoma. Clin. Transl. Gastroenterol. 12 (8), e00395. Published 2021 Aug 13. 10.14309/ctg.0000000000000395 34388137 PMC8367066

[B12] Li X. Qian X. Peng L.-X. Jiang Y. Hawke D. H. Zheng Y. (2016). A Splicing Switch from Ketohexokinase-C to Ketohexokinase-A Drives Hepatocellular Carcinoma Formation. Nat. Cell Biol. 18 (5), 561–571. 10.1038/ncb3338 27088854 PMC4888794

[B13] Li Y. Burgman B. McGrail D. J. Sun M. Qi D. Shukla S. A. (2020). Integrated Genomic Characterization of the Human Immunome in Cancer. Cancer Res. 80 (21), 4854–4867. 10.1158/0008-5472.CAN-20-0384 32855206 PMC7642109

[B14] Li Y. Li X.-Y. Li L.-X. Zhou R.-C. Sikong Y. Gu X. (2020). S100A10 Accelerates Aerobic Glycolysis and Malignant Growth by Activating mTOR-Signaling Pathway in Gastric Cancer. Front. Cell Dev. Biol. 8, 559486. Published 2020 Nov 26. 10.3389/fcell.2020.559486 33324631 PMC7726224

[B15] Liu L. Chen X. Petinrin O. O. Zhang W. Rahaman S. Tang Z.-R. (2021). Machine Learning Protocols in Early Cancer Detection Based on Liquid Biopsy, A SurveyLife 11 (7), 638. PMC8308091. 10.3390/life11070638.PMID:34209249 PMC8308091 34209249

[B16] Lux A. Kahlert C. Grützmann R. Pilarsky C. (2019). c-Met and PD-L1 on Circulating Exosomes as Diagnostic and Prognostic Markers for Pancreatic Cancer. Int. J. Mol. Sci. 20 (13), 3305. Published 2019 Jul 5. 10.3390/ijms20133305 PMC6651266 31284422

[B17] Mayes P. A. Degenhardt Y. Y. Wood A. Toporovskya Y. Diskin S. J. Haglund E. (2013). Mitogen-activated Protein Kinase (MEK/ERK) Inhibition Sensitizes Cancer Cells to Centromere-Associated Protein E Inhibition. Int. J. Cancer 132 (3), E149–E157. 10.1002/ijc.27781 22948716 PMC4706358

[B18] McGuire S. (2016). World Cancer Report 2014. Geneva, Switzerland: World Health Organization, International Agency for Research on Cancer, WHO Press, 2015. Adv. Nutr. 7 (2), 418–419. Published 2016 Mar 15. 10.3945/an.116.012211 26980827 PMC4785485

[B19] Morrison A. H. Byrne K. T. Vonderheide R. H. (2018). Immunotherapy and Prevention of Pancreatic Cancer. Trends Cancer 4 (6), 418–428. 10.1016/j.trecan.2018.04.001 29860986 PMC6028935

[B20] Piccolo B. D. Graham J. L. Kang P. Randolph C. E. Shankar K. Yeruva L. (2021). Progression of Diabetes Is Associated with Changes in the Ileal Transcriptome and Ileal‐colon Morphology in the UC Davis Type 2 Diabetes Mellitus Rat. Physiol. Rep. 9 (22), e15102. 10.14814/phy2.15102 34806320 PMC8606862

[B21] Qin Y. Hu Q. Ji S. Xu J. Dai W. Liu W. (2019). Homeodomain‐interacting Protein Kinase 2 Suppresses Proliferation and Aerobic Glycolysis via ERK/cMyc axis in Pancreatic Cancer. Cell Prolif. 52 (3), e12603. 10.1111/cpr.12603 30932257 PMC6536454

[B22] Quinn W. J. 3rd Jiao J. TeSlaa T. Stadanlick J. Wang Z. Wang L. (2020). Lactate Limits T Cell Proliferation via the NAD(H) Redox State. Cell Rep. 33 (11), 108500. 10.1016/j.celrep.2020.108500 33326785 PMC7830708

[B23] Schizas D. Charalampakis N. Kole C. Economopoulou P. Koustas E. Gkotsis E. (2020). Immunotherapy for Pancreatic Cancer: A 2020 Update. Cancer Treat. Rev. 86, 102016. 10.1016/j.ctrv.2020.102016 32247999

[B24] Seo A. Walsh T. Lee M. K. Ho P. A. Hsu E. K. Sidbury R. (2016). FAM111B Mutation Is Associated with Inherited Exocrine Pancreatic Dysfunction. Pancreas 45 (6), 858–862. 10.1097/MPA.0000000000000529 26495788 PMC4841754

[B25] Shang N. Wang H. Bank T. Perera A. Joyce C. Kuffel G. (2019). Focal Adhesion Kinase and β‐Catenin Cooperate to Induce Hepatocellular Carcinoma. Hepatology 70 (5), 1631–1645. 10.1002/hep.30707 31069844 PMC6819211

[B26] Shen N. Korm S. Karantanos T. Li D. Zhang X. Ritou E. (2021). DLST-dependence Dictates Metabolic Heterogeneity in TCA-Cycle Usage Among Triple-Negative Breast Cancer. Commun. Biol. 4 (1), 1289. Published 2021 Nov 16. 10.1038/s42003-021-02805-8 34785772 PMC8595664

[B27] Wang H. Rao B. Lou J. Li J. Liu Z. Li A. (2020). The Function of the HGF/c-Met Axis in Hepatocellular Carcinoma. Front. Cell Dev. Biol. 8, 55. Published 2020 Feb 7. 10.3389/fcell.2020.00055 32117981 PMC7018668

[B28] Wang M.-D. Wang N.-Y. Zhang H.-L. Sun L.-Y. Xu Q.-R. Liang L. (2021). Fatty Acid Transport Protein-5 (FATP5) Deficiency Enhances Hepatocellular Carcinoma Progression and Metastasis by Reprogramming Cellular Energy Metabolism and Regulating the AMPK-mTOR Signaling Pathway. Oncogenesis 10 (11), 74. Published 2021 Nov 12. 10.1038/s41389-021-00364-5 34772914 PMC8589992

[B29] Wu E. Q. Lin C.-T. Zhu L.-M. Tang Z. R. Jie Y.-W. Zhou G.-R. (2021). Fatigue Detection of Pilots' Brain through Brains Cognitive Map and Multilayer Latent Incremental Learning Model. IEEE Trans. Cybern. 2021, 1–13. Epub ahead of print. PMID: 33961575. 10.1109/TCYB.2021.3068300 33961575

[B30] Xie M. Fu X.-g. Jiang K. (2021). Notch1/TAZ axis Promotes Aerobic Glycolysis and Immune Escape in Lung Cancer. Cell Death Dis. 12 (9), 832. Published 2021 Sep 4. 10.1038/s41419-021-04124-6 34482375 PMC8418606

[B31] Yang C. Zhu S. Yang H. Deng S. Fan P. Li M. (2019). USP44 Suppresses Pancreatic Cancer Progression and Overcomes Gemcitabine Resistance by Deubiquitinating FBP1. Am. J. Cancer Res. 9 (8), 1722–1733. Published 2019 Aug 1. 31497353 PMC6726996

[B32] Ye Z. Zhu Z. Xie J. Feng Z. Li Y. Xu X. (2020). Hsa_circ_0000069 Knockdown Inhibits Tumorigenesis and Exosomes with Downregulated Hsa Circ 0000069 Suppress Malignant Transformation via Inhibition of STIL in Pancreatic Cancer. Int. J. Nanomed. 15, 9859–9873. 10.2147/IJN.S279258 PMC7732169 33324055

[B33] Zhong C. Li P. Argade S. Liu L. Chilla’ A. Liang W. (2020). Inhibition of Protein Glycosylation Is a Novel Pro-angiogenic Strategy that Acts via Activation of Stress Pathways. Nat. Commun. 11 (1), 6330. Published 2020 Dec 10. 10.1038/s41467-020-20108-0 33303737 PMC7730427

[B34] Zhuang H. Zhou Z. Ma Z. Li Z. Liu C. Huang S. (2020). Characterization of the Prognostic and Oncologic Values of ITGB Superfamily Members in Pancreatic Cancer. J. Cell. Mol. Med. 24 (22), 13481–13493. 10.1111/jcmm.15990 33073486 PMC7701563

