# Supplementary material for: Combining Single-Cell and Transcriptomic Data Revealed the Prognostic Significance of Glycolysis in Pancreatic Cancer
Source: Front Genet. 2022 Jul 5;13:903783. doi: 10.3389/fgene.2022.903783 (PMC9294390; doi:10.3389/fgene.2022.903783)
Supplement: Supplementary file 2 [file Table1.DOCX]

| Variables | TCGA Corhort | AU Corhort | CA Corhort |
| --- | --- | --- | --- |
| Gender  male  female  Age  <=65  >65  T  T1  T2  T3  T4  TX  unknown  N  N0  N1  NX  unknown  M  M0  M1  MX  Stage  Stage I  Stage II  Stage III  Stage IV  unknown | 96(54.5%)  80(45.5%)  81(46.0%)  95(54.0%)  7(4.0%)  24(13.6%)  140(79.5%)  3(1.7%)  1(0.6%)  1(0.6%)  49(27.8%)  122(69.3%)  4(2.2%)  1(0.6%)  79(44.9%)  4(2.2%)  93(52.8%)  21(11.9%)  145(82.4%)  3(1.7%)  4(2.2%)  3(1.7%) | 40(50%)  40(50%)  34(42.5%)  46(57.5%) | 95(54.9%)  78(45.1%)  82(47.4%)  91(52.6%)  55(31.8%)  89(51.4%)  8(4.6%)  21(12.1%) |
