# Supplementary figures and images for: Combining Single-Cell and Transcriptomic Data Revealed the Prognostic Significance of Glycolysis in Pancreatic Cancer
Source: Front Genet. 2022 Jul 5;13:903783. doi: 10.3389/fgene.2022.903783 (PMC9294390; doi:10.3389/fgene.2022.903783)

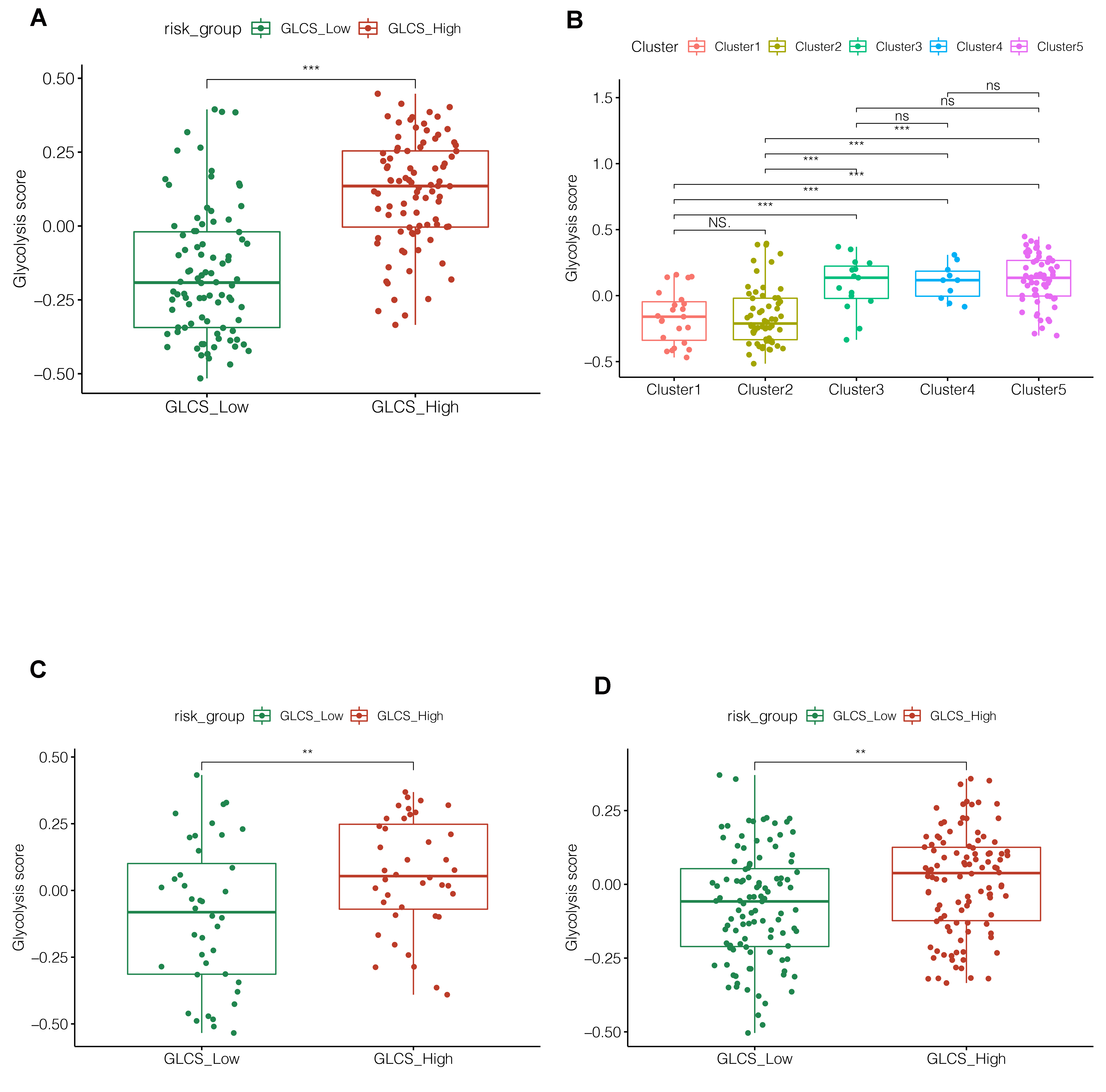

Supplement: Supplementary file 3 [file Image2.TIF]

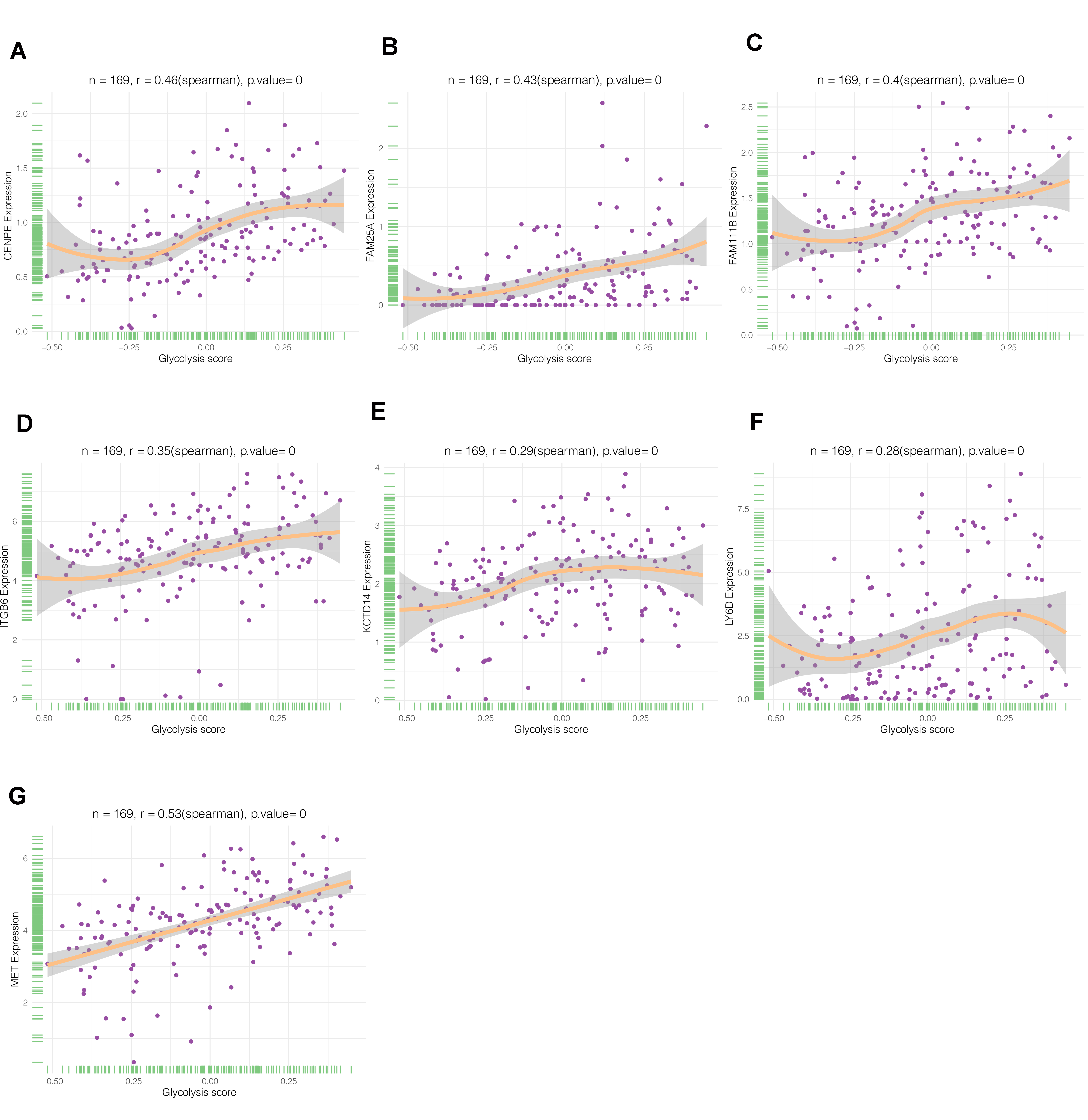

Supplement: Supplementary file 4 [file Image1.TIF]
