# Supplementary material for: Combining Single-Cell and Transcriptomic Data Revealed the Prognostic Significance of Glycolysis in Pancreatic Cancer
Source: Front Genet. 2022 Jul 5;13:903783. doi: 10.3389/fgene.2022.903783 (PMC9294390; doi:10.3389/fgene.2022.903783)
Supplement: Supplementary file 5 [file Table2.DOCX]

| MET(Forward) | TGGGCACCGAAAGATAAACCT |
| --- | --- |
| MET(Reverse) | TCTCGGACTTTGCTAGTGCC |
| FAM25A(Forward) | CGTGGAAGAAGTGGTGAAGGA |
| FAM25A(Reverse) | GACTCTCTGCTGCATGGGTG |
| LY6D(Forward) | CTCCTGGCCGTCATCTTAGC |
| LY6D(Reverse) | GACCTGGTCCCAGACTTTCG |
| FAM111B(Forward) | GGCACTGACGGGCACTT |
| FAM111B(Reverse) | TCTGCTTCATGACAGTATCCTTGA |
| ITGB6(Forward) | AGTTTCTTTTCAAAGCAGTCAAGA |
| ITGB6 (Reverse) | GGTCCAATAAGCAGGCAGTC |
| CENPE(Forward) | TACTGCTCTCCAGTTTGCCAG |
| CENPE (Reverse) | TTTTCCATTGCCTGAGCCCG |
| KCTD14(Forward) | CCTTGCGAACTGATTCTGTGG |
| KCTD14(Reverse) | GCCAATGTCACCCCCTGA |
